# Failed tracheal intubation in primary health care

**DOI:** 10.4102/safp.v64i1.5532

**Published:** 2022-10-24

**Authors:** Indiran Govender, Doudou K. Nzaumvila, Olga M. Maphasha

**Affiliations:** 1 Department Family Medicine and Primary Health Care, Faculty of Health Sciences, Sefako Makgatho Health Sciences University, Pretoria, South Africa; 2 Department Family Medicine, Faculty of Health Sciences, Sefako Makgatho Health Sciences University, Pretoria, South Africa; 3 Department of Family Medicine, Faculty of Medicine, University of Pretoria, Pretoria, South Africa

**Keywords:** failed intubation, anaesthesia, airway management, primary health care, Mallampati classification, video laryngoscope, hypoxia, hypotension

## Abstract

Tracheal intubation in primary health care is a necessary skill and usually one that is necessary for appropriate emergency management of unstable patients. Primary care practitioners may not have an anaesthetist or critical care doctor available to help them in these emergencies and must manage these patients themselves. Often tracheal intubation may fail because of multiple possible factors and a different course of action may be needed to minimise the potential for harm to the patient. The primary care professional or family physician will have to manage this failed intubation. Primary health care facilities providing obstetric services must have guidelines and appropriate equipment for management of airway problems. This article will explore reasons for the failure of tracheal intubation and how this can be managed.

## Introduction

Failure to achieve a successful tracheal intubation in a maximum of three attempts, regardless of the techniques used, is defined as failed tracheal intubation.^1^ Firstly, one would like to note the difference between difficult intubation and, secondly, the new concept of physiological failed intubation. Tracheal intubation is considered difficult if more than one attempt at optimised laryngoscopy and tracheal tube passage is made, a more experienced healthcare worker is required or a change in technique and/or device is made.^1^ Most recently, the American Society of Anaesthesiologists Practice Guidelines for Management of the Difficult Airway defined both difficult and failed tracheal intubation as a tracheal intubation that necessitates multiple attempts or tracheal intubation that fails after multiple attempts.^2^ This concept mainly emphasises the anatomical difficulties of visualising the glottic opening or inserting the tracheal tube through the vocal cords.^3,4^ However, in a very ill patient (or even in healthy patients), physiological changes prior to intubation may compromise the gas exchange after the endotracheal tube is placed; this constitutes a physiological failed intubation.^3,4^

Most tracheal intubations are indicated for airway protection during surgical procedures or airway protection for trauma patients rather than respiratory failure.^5^ In primary health settings, patients requiring tracheal intubations are those who are in need of general anaesthesia (GA) for obstetrics and gynaecology (laparotomy for ectopic pregnancy, sometimes C-section) for trauma and general surgery. Data from an obstetrics article revealed that although the number of deaths related to failed tracheal intubation has lessened considerably over the last three decades, the incidence of failed intubation has stayed roughly steady at between 1:224 and 1:300.^6^ According to the data, not all occurrences seem to be predictable, nor are they all preventable.^7^ However, as failed intubation is an emergency and difficult airways are anticipated, early recognition followed by adequate management as soon as humanly possible are paramount to avoid complications, because airway management–related morbidity is still being reported in closed legal claims.^8,9^

Junior doctors or doctors with limited anaesthetic training and experience are responsible for providing obstetric anaesthesia in the district hospitals.^10^ Even though tracheal intubation is a core skill for medical interns, training is provided for two months that leads to the lack of this essential skill when they are community service doctors working in district hospitals.^10^

According to the report from a survey conducted in the United Kingdom in 1995, a supraglottic device laryngeal mask airway was used by 72% of anaesthetists in obstetric anaesthesia to maintain oxygenation when tracheal intubation and face-mask ventilation failed.^7,11^ The choice of a supraglottic device to use for airway rescue management will be determined by the clinical situation, device availability and the clinician’s experience.^12^

## Factors associated with failed tracheal intubation

Considering factors associated with failed intubation in primary health care, one should differentiate factors related to the general population and those related to obstetric population. In the general population, the age group of 40–59 years and body mass index (BMI) > 30 kg/m^2^ were reported to be associated with failed intubation,^7^ whereas in the obstetric population, factors reported were demographics of the obstetric patients (obesity, maternal age and pre-existing maternal morbidity) and anaesthetic factors. The most common type of anaesthesia for a C-section is spinal anaesthesia, and most clinicians do not have the ability to give GA and protect the airway when spinal anaesthesia fails.^10,11^ For some specific conditions and spinal failure, obstetric GA is recommended.

**FIGURE 1:**
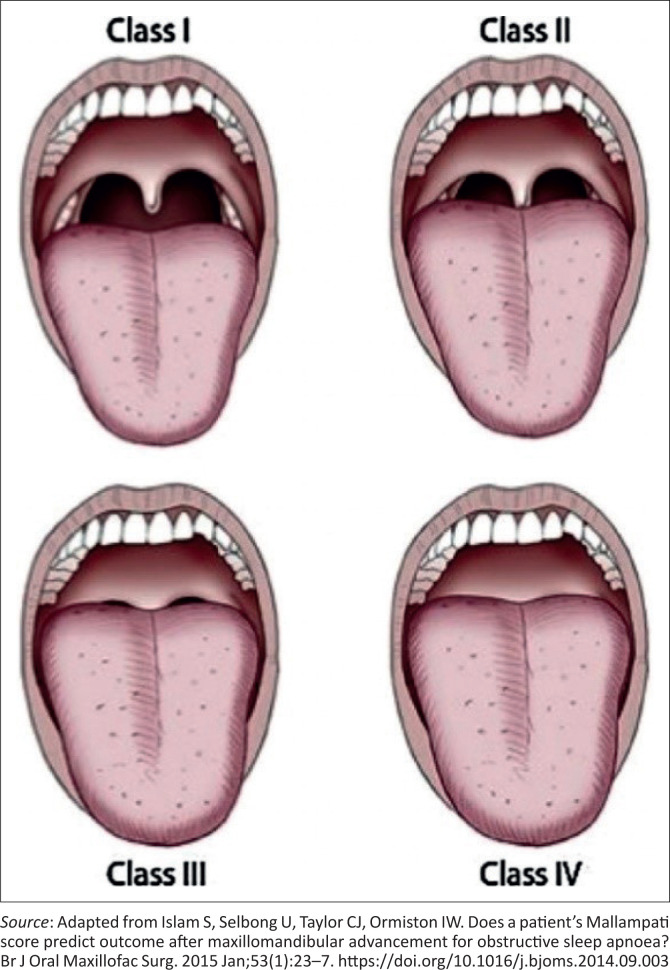
Mallampati classification: Class I: fully visible uvula and soft palate; Class II: hard and soft palate and upper portion of uvula are visible; Class III: soft and hard palate and base of uvula are visible; Class IV: only hard palate visible.

Video laryngoscopy has been shown to lower the incidence of failed intubation by providing a better view of the glottis, but it is not readily available in primary care settings.^6,13^ Video laryngoscopes provide a better view of the glottis; while failure to intubate in general has been documented to occur primarily in planned operations,^7^ some evidence suggests that failed intubations in obstetric patients occur in emergencies and after normal working hours, with the rationale being that airway assessment could not be performed.^14^ Mucosal airways easily get more vascular and oedematous as a result of pregnancy-related venous congestion, which can be exacerbated by eclampsia, intravenous fluids and oxytocin given during labour and sometimes Valsalva movements or pushing attempts. Changes in the mucosa results in increasing Mallampati’s score as pregnancy progresses (Figure 1^15^).^16^ Classification includes four grades (1, 2, 3 and 4) depending on visibility of the soft palate, thereby increasing the incidence of failed intubation in obstetrics.^17^ Other predictors of difficult intubation can be assessed using the ‘look – evaluate – Mallampati – obstruction – neck mobility’ (LEMON) classification.^18^

It is also worth noting that women about to give birth have a lower functional residual capacity, reduced cardiac tone, as well as slowed stomach emptying caused by labour pain and opioid treatment, all of which put together have a contributing effect on failed intubation.^6^ Increased gastric insufflation, trauma to the posterior pharynx, increased blood and secretions in the airway and oedema to the subglottic structures result from failed intubation, thereby making subsequent intubation more difficult.^19^

Besides anatomical changes mentioned here, there are some physiological changes that can lead to failure of optimised gas exchange after intubation.^3,4^

### Hypoxia

Critically ill patients suffering from hypoxemic respiratory failure and obese patients are reported more likely to experience rapid desaturation during intubation, which can lead to haemodynamic instability, hypoxic brain injury and potentially cardiopulmonary arrest. Two different types of hypoxia have been identified. Hypoxic hypoxia or hypoxemic respiratory failure (type I) caused by any etiopathogenesis that adversely affects optimal alveolar-capillary gas exchange (pneumonia, acute respiratory distress syndrome and cardiogenic or noncardiogenic pulmonary oedema) and hypemic hypoxia or hypercapnic respiratory failure (type II), caused by significantly reduced alveolar ventilation or an increase in dead space. However, apart from assessing predicted anatomic difficulties, identifying patients at risk for desaturation during intubation and maximising safe apnoea time by adequate and adapted preoxygenation should be the objective.^3,4^

### Hypotension

Post-intubation hypotension (PIH) is defined as a decrease in systolic blood pressure (SBP) to ≤ 90 mmHg or ≥ 20% from a baseline, a decrease in mean arterial pressure (MAB) to ≤ 65 mmHg or the initiation of vasopressors within the 30 min following intubation.^20^ It affects up to 25% of emergency department (ED) intubations, which are associated with high mortality and prolonged intensive care unit (ICU) care.^20^ According to current evidence, shock index of ≥ 80% (heart rate/SBP) is the best predictor of PIH (sensitivity of 67% and specificity of 80%) as compared with other predictors found in literature such as age, acute respiratory failure and chronic renal failure.^20^

### Severe metabolic acidosis

A critically ill patient with severe metabolic acidosis compensates by increasing minute ventilation. The so-called safe apnoeic period during intubation may be harmful, even if brief, as it can result in decrease of pH and a high risk of haemodynamic worsening after intubation.^3^

### Right ventricular failure

Any patient with right ventricular failure (RVF) is at risk of cardiorespiratory collapse post-intubation. Some conditions increase the burden of the right ventricle, such as chronic pulmonary hypertension from lung or left ventricular disease, pulmonary arterial hypertension or acute pulmonary embolism. When invasive mechanical ventilation is added to the decreased preload and increased right ventricular afterload, cardiac arrest is likely common.^3^

## Management of failed intubation in primary care

**FIGURE 2:**
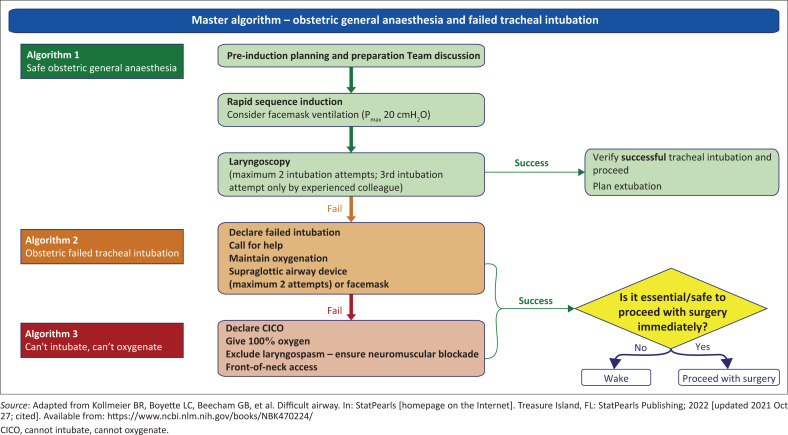
Obstetric general anaesthesia and failed tracheal intubation.

**FIGURE 3:**
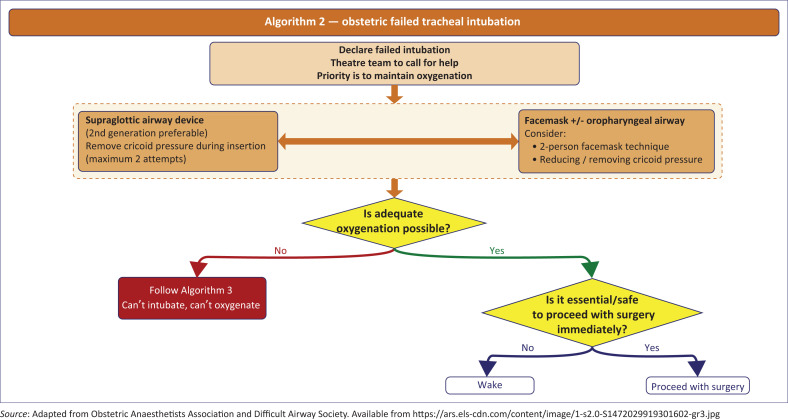
Obstetric failed tracheal intubation.

The first step in the management of a difficult airway is identification. It is vital that a call for help should be made immediately if a difficult airway is identified (Figure 2 and Figure 3)^6,18^ with a clear procedure of how to contact a senior personnel and referral route.^1,6,12^ Follow guidelines, for example, Difficult Airway Society guidelines to assist in decision-making during an emergency, as they include steps to assist the team in making the correct decisions, limiting number of airway intervention attempts, encouraging declaration of failure by placing a supraglottic airway device even when face-mask ventilation is possible and explicitly recommending a time to stop and think about how to proceed (Figure 3).^1,6,12^ The focus is to maintain oxygenation and prevent possible aspiration and awareness.^5,12^

As soon as correct tracheal tube placement cannot be confirmed after two attempts and a third attempt by an experienced practitioner, failed intubation should be declared.^6,12^ The use of a tracheal tube introducer has been extensively studied as an adjunct to direct laryngoscopy (DL). For tracheal intubation, a malleable stylet and bougie should be placed in the endotracheal tubes for all obstetric intubations.^12,16^ External laryngeal pressure should be applied if poor view with proper positioning of the patient is obtained during attempted DL (Figure 3).^1,12^ View can be improved by applying external laryngeal manipulation. Use the anaesthetist’s right hand or backward, upward and rightward pressure (BURP) on the thyroid cartilage.^12^ Tracheal intubation cannot be confirmed if correct placement of the tube between vocal cords was not visualised, with no bilateral chest expansion and absence of exhaled carbon dioxide (CO_2_) on capnography.^12^

Once failed intubation has been confirmed, use an oropharyngeal airway, four-handed facemask technique and the ‘sniffing’ position to optimise ventilation, reducing or removing cricoid pressure.^6,12,21^ Supraglottic airway devices (SADs) may be used as an option to facemask ventilation (Figure 3).^12,15,22^

**FIGURE 4:**
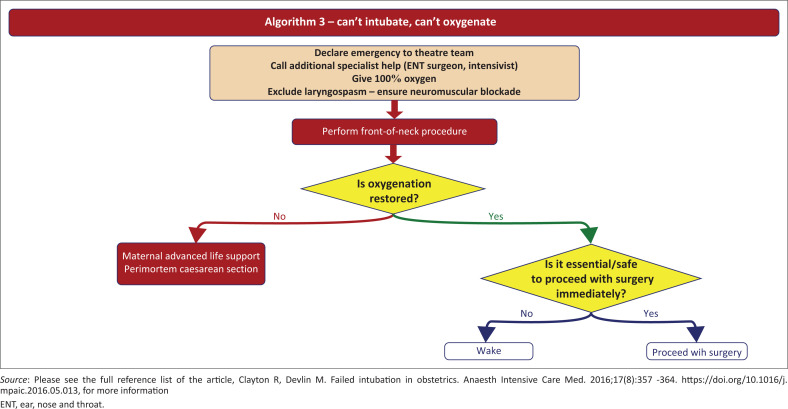
‘Can’t intubate, can’t oxygenate’.

**FIGURE 5:**
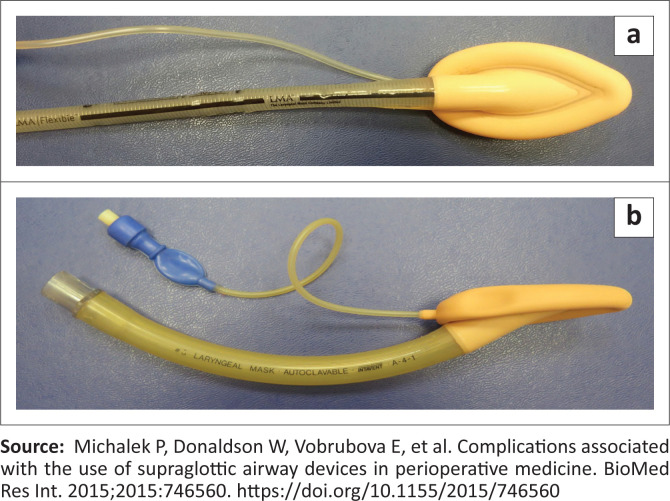
Laryngeal mask airway devices (first generation).

Supraglottic airway devices are positioned outside of the larynx. They are less invasive tools used for airway management in anaesthesia.^22^ Since the late 1990s, there has been a gradual increase in the use of SAD to continue anaesthesia when failed intubation is declared.^16,22^ Supraglottic airway devices are commonly classified into first-generation devices, containing only a breathing lumen (Figure 4^6,16^), and second-generation SADs (Figure 5^23^), which possess an additional channel for drainage of gastric contents.^12,22^ Second-generation devices are recommended as better rescue devices after failed tracheal intubation, even though they do not provide complete protection against aspiration (Figure 5).^6^ These devices have features such as drain tubes or compartments for mitigating risk of aspiration to manage regurgitated content,^22^ and they also provide a better airway seal for positive pressure ventilation.^15^

Supraglottic airway device placement requires less expertise and time for insertion and is associated with fewer complications, as compared with tracheal tube insertion.^12,22^ It is recommended that all clinicians in a position to give anaesthesia should be trained to use and have access to second-generation SADs.^12^

Supraglottic airway devices such as laryngeal mask airway devices can be successfully placed on the first attempt (Figure 2).^6,23^ Even though application of cricoid pressure is controversial, it is used in obstetric anaesthesia for the purpose of reducing regurgitation risk.^1^ It must be released during the insertion of (preferably) a second-generation SAD with a gastric drain.^3^ Repeated insertion attempts increase the likelihood of airway trauma and may delay the decision to accept failure to move on to an alternative technique so that oxygenation is maintained.^6,23^ It is crucial that the practitioner is familiar with their use.^6^

**FIGURE 6:**
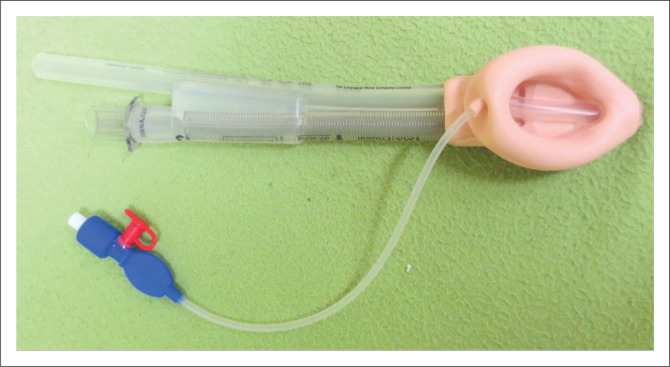
ProSeal laryngeal mask airway devices (second generation).

Difficult face mask ventilation of the unconscious or induced patient before or between tracheal intubation attempts should be addressed with an appropriate response, including placement of an appropriately sized oropharyngeal and nasopharyngeal airway, use of a two-handed mask hold and exaggerated head extension, unless contraindicated.^1,6^ Optimise airway position and attempt airway manoeuvres such as chin lift or jaw thrust if difficulty is encountered.^12^ Face mask ventilation may reduce the risk of significant desaturation.^6^ Emergency front of neck access (FONA) can be used if clinical deterioration and worsening saturation happens (Figure 6).^16^

Delay in delivery during management of a difficult airway at caesarean section might further potentiate the effects of maternal physiological derangement. The greatest delay in delivery will be incurred if the woman is awakened after failed intubation.^16^

**FIGURE 7:**
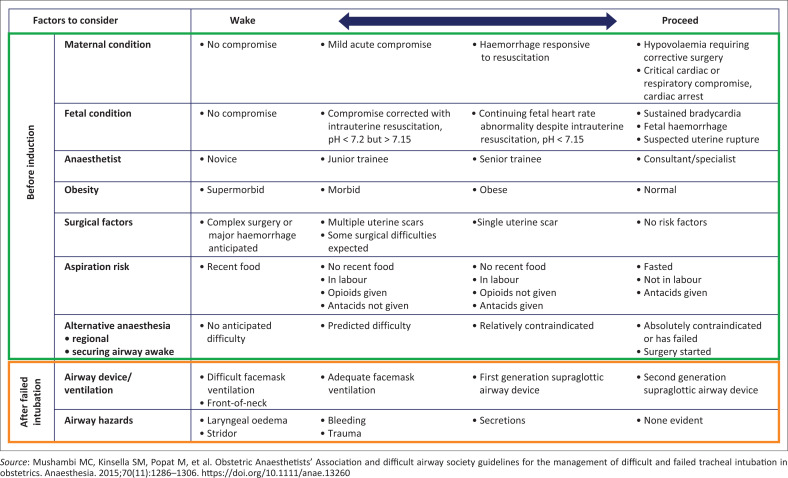
Wake or proceed with surgery, factors to consider.

A decision needs to be made whether it is safe or necessary to continue with the operation (Figure 6).^16^ This decision can be made with the surgeon pre-operatively during preparation, in case there is a failed intubation, following the World Health Organization surgical checklist.^6,16^ It is influenced by factors related to woman, foetus, staff and clinical situation (Figure 7).^16^ If the surgery is not urgent, the safest option is to wake the patient up. The following features related to the urgency of the procedure – the condition of the foetus, the condition of the mother, anaesthetic experience – must be taken into consideration,^16^ not forgetting local practice within the hospital. Oxygenation must be maintained throughout.^15^ Proceed with surgery if there is adequate airway or ventilation, and further intubation attempts are discouraged unless a new factor presents that significantly increases the chance of success, or there is an indication for prolonged airway control.^16^

When it comes to physiological failed intubation, in the case of a critically hypoxic patient, apnoeic oxygenation should always come just after standard practice of preoxygenation.^3^ Using a wide-bore nasal cannula, a high-flow nasal cannula or conventional nasal prongs helps to extend the safe apnoeic period.^3^ Should shunt physiology be caused by atelectasis or alveolar filling from pneumonia, acute respiratory distress syndrome (ARDS) or pulmonary oedema be suspected, nasal intermittent positive pressure ventilation (NIPPV) or supraglottic airways can be used^24^ with the aid of analgesia, anxiolysis or delayed sequence intubation (DSI) if indicated; however, use of ketamine should be cautious because of the risk of cardiac arrest.^3^ Tracheal intubation should be delayed in case of hypotension and shock index ≥ 0.8 to give time to aggressive volume resuscitation if the patient is likely to be a volume responder.^3^ If a patient is unresponsive to volume resuscitation, a norepinephrine infusion should be considered or vasopressor bolus if there is no time for pre-intubation resuscitation. Because many induction drugs have negative haemodynamic effects and some can cause peri-intubation hypotension, one should exercise extreme caution in the product to be used. Etomidate, for example, a nonbenzodiazepine sedative, is basically haemodynamically neutral^25^ as compared with benzodiazepine agents, which have a sympatholytic effect, or propofol, which causes myocardial depression and a decline in vascular tone.^26^ Some authors suggested the use of ketamine, considering its sympathomimetic properties, even though there have been reports of cardiac arrest following ketamine administration.^27^ To prevent physiologically unsuccessful intubation in patients with severe metabolic acidosis, other forms of support, such as NIPPV, should be used to try to correct the acidosis first. Even after intubation, the patient should be allowed to maintain their own high minute ventilation.^3^

## Conclusion

The most common complication of anaesthesia in primary health settings is failed intubation. Early identification and management are crucial in managing all patients from EDs and theatres. Video laryngoscopy and SADs are useful tools to have during management of difficult and failed intubation, only if the primary health care practitioner is familiar with them. Most primary health care setting theatres do not have access to video laryngoscopy. It is important for the theatre team to follow local guidelines when faced with difficult intubation.

## References

[1] Law JA, Broemling N, Cooper RM, et al. The difficult airway with recommendations for management – Part 1 – Difficult tracheal intubation encountered in an unconscious/induced patient. Can J Anaesth. 2013;60(11):1089–1118. 10.1007/s12630-013-0019-3 24132407 PMC3825644

[2] Jeffrey L, Apfelbaum CA, Hagberg RT, et al. American society of anesthesiologists practice guidelines for management of the difficult airway. Anesthesiology. 2022;136:31–81. 10.1097/ALN.0000000000004002 34762729

[3] Mosier JM, Joshi R, Hypes C, Pacheco G, Valenzuela T, Sakles JC. The physiologically difficult airway. West J Emerg Med. 2015;16(7):1109–1117. 10.5811/westjem.2015.8.27467 26759664 PMC4703154

[4] Myatra SN, Divatia JV, Brewster DJ. The physiologically difficult airway: An emerging concept. Curr Opin Anaesthesiol. 2022;35(2):115–121. 10.1097/ACO.0000000000001102 35165233

[5] Zewdie A, Tagesse D, Alemayehu S, Getachew T, Sultan M. The success rate of endotracheal intubation in the emergency department of Tertiary Care Hospital in Ethiopia, one-year retrospective study. Emerg Med Int. 2021;2021:9590859. 10.1155/2021/9590859 33828865 PMC8004359

[6] Clayton R, Devlin M. Failed intubation in obstetrics. Anaesth Intensive Care Med. 2016;17(8):357–364. 10.1016/j.mpaic.2016.05.013

[7] Endlich Y, Lee J, Culwick MD. Difficult and failed intubation in the first 4000 incidents reported on webAIRS. Anaesth Intensive Care. 2020;48(6):477–487. 10.1177/0310057X20957657 33203219

[8] Law JA, Duggan LV, Asselin M, et al. Canadian Airway Focus Group consensus-based recommendations for management of the difficult airway: Part 2: Planning and implementing safe management of the patient with an anticipated difficult airway. Can J Anesth. 2021;68:1405–1436. 10.1007/s12630-021-02008-z 34105065 PMC8186352

[9] Crosby ET, Duggan LV, Finestone PJ, Liu R, De Gorter R, Calder LA. Anesthesiology airway-related medicolegal cases from the Canadian Medical Protection Association. Can J Anaesth. 2021;68(2):183–195. 10.1007/s12630-020-01846-7 33200320 PMC7668407

[10] Tomlinson JMB, Bishop DG, Hofmeyr R, et al. The incidence and predictors of hypoxaemia during induction of general anaesthesia for caesarean delivery in two South African hospitals: A prospective, observational, and dual-centre study. S Afr J Anaesth Analg. 2020;26(4):183–187. 10.36303/SAJAA.2020.26.4.2345

[11] Frerk C, Mitchel CS, McNarry AF, Menodnca C. Difficulty airway society 2015 guidelines for management of unanticipated difficult intubation in adults. Br J Anaesth. 2015;117(4):529–530. 10.1093/bja/aew279 PMC4650961 26556848

[12] Metdiev Y, Mushanbi M. Supraglottic airway devised for caesarean delivery under general anaesthesia: For all, for none, or for some? Br J Anaesth. 2020;125(1):e7–e11. 10.1016/j.bja.2020.02.012 32197776

[13] Makoko UM, Modiba LM, Nzaumvila DK. Satisfaction with spinal anaesthesia for caesarean section at Tembisa Hospital, South Africa: A cross-sectional study. S Afr Fam Pract. 2018;61(2):39–47. 10.1080/20786190.2018.1531585

[14] Lewis SR, Butler AR, Parker J, Cook TM, Smith AF. Videolaryngoscopy versus direct laryngoscopy for adult patients requiring tracheal intubation. Cochrane Database Syst Rev. 2016;(11):CD011136. 10.1002/14651858.CD011136.pub2 27844477 PMC6472630

[15] Islam S, Selbong U, Taylor CJ, Ormiston IW. Does a patient’s Mallampati score predict outcome after maxillomandibular advancement for obstructive sleep apnoea? Br J Oral Maxillofac Surg. 2015 Jan;53(1):23–7. 10.1016/j.bjoms.2014.09.003 25266137

[16] Hawthorne L, Wilson R, Lyons G, Dresner M. Failed intubation revisited: A 17-year experience in a teaching maternity unit. Br J Anaesth. 1996;76:680–684. 10.1093/bja/76.5.680 8688269

[17] Mushambi MC, Kinsella SM, Popat M, et al. Obstetric Anaesthetists’ Association and difficult airway society guidelines for the management of difficult and failed tracheal intubation in obstetrics. Anaesthesia. 2015;70(11):1286–1306. 10.1111/anae.13260 26449292 PMC4606761

[18] Msheila DB, Ogbalu-Nwasor EO, Isamade ES. Use of the ‘L-E-M-O-N’ score in predicting difficult intubation in Africans. Niger J Basic Clin Sci. 2018;15(1):17–23. 10.4103/njbcs.njbcs_25_16

[19] Shivashankar A, Rafappa G, Rath P, et al. Change in Mallampati score during pregnancy, labour and post labour: An observational study. Sri Lankan J Anaesthesiol. 2021;29(1):13–17. 10.4038/slja.v29i1.8653

[20] Althunayyan SM. Shock index as a predictor of post-intubation hypotension and cardiac arrest: A review of the current evidence. Bull Emerg Trauma. 2019;7(1):21–27. 10.29252/beat-070103 30719462 PMC6360014

[21] Kollmeier BR, Boyette LC, Beecham GB, et al. Difficult airway. In: StatPearls [homepage on the Internet]. Treasure Island, FL: StatPearls Publishing; 2022 [updated 2021 Oct 27; cited]. Available from: https://www.ncbi.nlm.nih.gov/books/NBK470224/29261859

[22] Michalek P, Donaldson W, Vobrubova E, et al. Complications associated with the use of supraglottic airway devices in perioperative medicine. BioMed Res Int. 2015;2015:746560. 10.1155/2015/746560 26783527 PMC4691459

[23] Doyle DJ, Hagberg CA, Crowley M. Supraglottic devices (including laryngeal mask airways) for airway management for anesthesia in adults [homepage on the Internet]. [cited 2022 Jun 14]. Available from: https://www.uptodate.com/contents/supraglottic-devices-including-laryngeal-mask-airways-for-airway-management-for-anesthesia-in-adults

[24] Heffner AC, Swords D, Kline JA, et al. The frequency and significance of postintubation hypotension during emergency airway management. J Crit Care. 2012;27(4):417.e9–e417.e13. 10.1016/j.jcrc.2011.08.011 22033053

[25] Morris C, Perris A, Klein J, et al. Anaesthesia in haemodynamically compromised emergency patients: Does ketamine represent the best choice of induction agent? Anaesthesia. 2009;64(5):532–539. 10.1111/j.1365-2044.2008.05835.x 19413824

[26] Ebert TJ, Muzi M, Berens R, et al. Sympathetic responses to induction of anesthesia in humans with propofol or etomidate. Anesthesiology. 1992;76:725–733. 10.1097/00000542-199205000-00010 1575340

[27] Strayer RJ, Nelson LS. Adverse events associated with ketamine for procedural sedation in adults. Am J Emerg Med. 2008;26(9):985–1028. 10.1016/j.ajem.2007.12.005 19091264

